# Antioxidative Activity of Soy, Wheat and Pea Protein Isolates Characterized by Multi-Enzyme Hydrolysis

**DOI:** 10.3390/nano11061509

**Published:** 2021-06-07

**Authors:** Chiung-Yueh Chang, Jinn-Der Jin, Hsiao-Li Chang, Ko-Chieh Huang, Yi-Fen Chiang, Mohamed Ali, Shih-Min Hsia

**Affiliations:** 1 School of Nutrition and Health Sciences, College of Nutrition, Taipei Medical University, Taipei 110, Taiwan; d507104001@tmu.edu.tw (C.-Y.C.); a910241@gmail.com (K.-C.H.); yvonne840828@gmail.com (Y.-F.C.); 2 GeneFerm Biotechnology Co., Ltd., Tainan 741, Taiwan; jin168@geneferm.com (J.-D.J.); gracechang@geneferm.com (H.-L.C.); 3 Clinical Pharmacy Department, Faculty of Pharmacy, Ain Shams University, 11566 Cairo, Egypt; mohamed.aboouf@pharma.asu.edu.eg; 4 Graduate Institute of Metabolism and Obesity Sciences, College of Nutrition, Taipei Medical University, Taipei 110, Taiwan; 5 School of Food and Safety, Taipei Medical University, Taipei 110, Taiwan; 6 Nutrition Research Center, Taipei Medical University Hospital, Taipei 110, Taiwan

**Keywords:** plant protein isolate, enzyme hydrolysis, characteristics, cell viability, antioxidant

## Abstract

Hydrolysis of protein by proteases produces small molecular weights (MWs) peptides as nanomaterials that are easily absorbed. This study investigated the physicochemical properties and antioxidant activity of three plant protein isolates (PIs) including soy, wheat and pea after multi-enzyme hydrolysis. The MWs, particle size and microstructure of PI hydrolysate (PIH) were determined by SDS-PAGE and MALDI-TOF-MS mass spectrometry, dynamic light scattering and transmission electron microscopy, respectively. Cell viability was determined in vitro using a mouse skeletal muscle cell line (C2C12) and crystal violet staining. The MWs and particle sizes of the three plant PIs were reduced after hydrolysis by three proteases (bromelain, Neutrase and Flavourzyme). The MWs of soy, wheat and pea PIH were 103.5–383.0 Da, 103.5–1146.5 Da and 103.1–1937.7 Da, respectively, and particle size distributions of 1.9–2.0 nm, 3.2–5.6 nm and 1.3–3.2 nm, respectively. All three plant PIHs appeared as aggregated nanoparticles. Soy PIH (100 μg/mL) provided better protection against H_2_O_2_-induced oxidative damage to C2C12 than wheat or pea PIH. In summary, soy PIH had the best antioxidant activity, and particle size than wheat PIH and pea PIH. Therefore, soy PIH might be a dietary supplement for healthy diet and medical applications.

## 1. Introduction

Plant-based proteins have potential health benefits [1,2,3], environmental friendliness [4] and low production costs [5], therefore they have gained increasing interest for researchers and consumers in recent years. Enzymatic hydrolysis can improve the physiological activity of proteins by converting them into peptides of different molecular sizes, charges and surface hydrophobic properties [6,7,8]. According to Sánchez and Vázquez [9], hydrolysis products of proteins exhibited higher physiological activities and could be used as antibacterial agents, angiotensin converting enzyme inhibitors, antioxidants and immunomodulatory agents. Of these, protein hydrolysates have better antioxidant activity than natural proteins due to their various properties, including acting as metal-ion chelators, hydrogen donors or oxygen quenchers to scavenge free radicals [10].

Particles with a size of 1–100 nm have specific physicochemical and biological activities [11] and can be used as nanocarriers and nanomedicines [12]. Nanotechnology is an emerging technology that is used to produce nanoparticles with critical research roles and applications. In general, protein nanoparticles can be produced through hydrolysis [13]. Proteins are commonly hydrolyzed by enzymatic hydrolysis, microbial fermentation and chemical hydrolysis [14]. Compared with chemical hydrolysis, enzymatic hydrolysis is more specific, which enables the production of desired hydrolysates through the selection of enzymes, thus retaining their nutritional value [14]. Enzymes can be classified as endopeptidases and exopeptidases depending on how they act on substrates. Endopeptidases start the hydrolysis action from the peptide bond inside the protein. Exopeptidases can be classified as aminopeptidases acting on the N-terminal α-amino group and carboxypeptidases acting on the C-terminal α-carboxyl group [15]. Bromelain (EC 3.4.22.32) is a cysteine endoprotease derived from pineapple stems that randomly hydrolyzes internal peptide bonds to breaks down proteins into peptides [16]. Neutrase (EC 3.4.24.28) is a neutral, zinc metallo endo-protease from *Bacillus amyloliquefaciens* with optimal activity at pH 5.5–7.5 and 30–55 °C [17]. Flavourzyme is sold as an industrial peptidase derived from *Aspergillus oryzae* and is mainly used to produce flavor-active compounds from various protein sources. Flavourzyme contains eight enzymes, including two aminopeptidases (leucine aminopeptidase A, EC 3.4.11; leucine aminopeptidase 2, EC 3.4.11), three endopeptidases (neutral protease 1, EC 3.4.24; neutral protease 2, EC 3.4.24.39; alkaline protease 1, EC 3.4.21.63), two dipeptidyl peptidases (dipeptidyl peptidase 5, EC 3.4.14; dipeptidyl peptidase 4, EC 3.4.14.5) and one amylase (α-amylase A type 3, EC 3.2.1.1) [18].

The gradual loss of skeletal muscle mass and strength with age leads to a decline in function, termed sarcopenia [19], which might be triggered by oxidative stress. Under aging conditions, the production of reactive oxygen species (ROS) is increased accompanied by a decrease in antioxidant capacity, which in turn induces oxidative stress that is essential for cell death. Under such conditions, the proliferation and differentiation abilities of satellite cells required for muscle repair and/or regeneration are reduced, leading to a progressive decline in muscle mass and function [20]. C2C12 myotubes, which exhibit the characteristics of normal myogenic cells, are a commonly used model to study muscle cell growth and differentiation. Salucci et al. [21] demonstrated that C2C12 myoblasts were sensitive to different chemical reagents and their mitochondrial permeability changed in response to various external stimuli, leading to the apoptosis of skeletal muscle cells.

In recent years, the antioxidant capacities of proteins derived from various plant sources including black soybean [22], chickpea protein [23], potato protein [24,25] and sweet potato protein [26] have been extensively studied. However, the physicochemical properties of plant PIH (soy, wheat and pea), as well as their antioxidant capacities in skeletal muscle cells have not been reported. We investigated the physicochemical properties and antioxidant activities of soy, wheat and pea proteins after multi-enzyme hydrolysis using bromelain, Neutrase and Flavourzyme. The MWs distribution, particle size and microstructure of the obtained PIH were determined and the antioxidant effects of PIH on skeletal muscle C2C12 cells were evaluated. The aim of this study was to investigate the physicochemical properties of plant PIH and their antioxidant activities in C2C12 murine myoblast cells.

## 2. Materials and Methods

### 2.1. Materials and Preparation Method

Soy PI was purchased from YUWANG ECO. (Shandong, China) and wheat PI and pea PI were purchased from Roquette (Lestrem, France). Plant PIH were prepared by the hydrolysis of plant PI using three proteases: bromelain (EC 3.4.22.32, Chappion Biotechnology, Chiayi, Taiwan), Neutrase (EC 3.4.24.28, Novozymes, Bagsvaerd, Denmark) and Flavourzyme (EC 3.4.11.1, Novozymes). Briefly, plant PI solution (100 mg plant PI /mL) was prepared by dissolving plant PI (1 kg) in distilled water (10 L) at 95 °C for a period of 1 h. Subsequently, three proteases including bromelain (1,000 CDU/mL), Neutrase (0.0024 AU-N/mL) and Flavourzyme (3.3 LAPU/mL) were added to the plant PI solution. The protease-containing plant PI solution was incubated at 45 °C for 24 h. Then, the hydrolyzed plant PI solution was heated at 95 °C for 1 h to inactivate the protease. The plant PI solution was then centrifuged at 9000× *g* at 4 °C for 10 min. Filtering the supernatant using a filter membrane (No. 1, pore size: 6 µm, Advantec Co. Ltd., Tokyo, Japan). Note that the proteases (bromelain, Neutrase^®^ and Flavourzyme^®^) were denatured during the heating and filtration process and were therefore not included in the plant PIH. Finally, the plant PIH solution was freeze-dried to obtain a powder that was kept in an airtight container at 25 °C before use.

### 2.2. MWs Analysis

The MWs distributions of plant PI and PIH were determined by sodium dodecyl sulfate-polyacrylamide gel electrophoresis (SDS-PAGE) as reported by Laemmli [27]. SDS-PAGE analysis was performed with a 12.5% separation gel and 5% stacking gel. Typically, 6 mg of the sample was added to 1 mL of buffer (0.02% bromophenol blue, 2% SDS, 5% β-mercaptoethanol, 10% glycerol and 70 mM Tris-HCl, pH 6.8). The mixture was well-mixed and placed on a heating plate at 95 °C for 7 min. Sample (10 μL) and protein marker (6 μL) were separately loaded into separate wells. Each sample contained 60 μg of protein. After the completion of electrophoresis, gels were stained with Coomassie Brilliant Blue R-250 and decolorized with 10% acetic acid until they appeared transparent with banding. Finally, the stained gels were scanned using an Epson perfection image scanner (Epson America Inc., Long Beach, CA, USA). In addition, the MWs of plant PIH were analyzed by matrix-assisted laser desorption/ionization time-of-flight mass spectrometry (MALDI-TOF-MS) using a mass spectrometer (Autoflex III, Bruker Daltonik, Bremen, Germany). The MWs measurement range was 0–2000. All measurements were repeated three times (*n* = 3).

### 2.3. Particle Size Analysis

The particle size distribution (PSD) of plant PIH was determined using a 90Plus Nanoparticle Size Analyzer (Brookhaven Instruments, Holtsville, NY, US). The particle sizes of the samples were analyzed using the method proposed by Win and Feng [28]. A sample solution (0.5% *w/v*) was prepared by dissolving the required amount of sample in secondary water (dd water), and stirred at 25 °C for 20 min. Then, the sample solution was diluted 10 times with secondary water and centrifuged at 12,000× g for 20 min at 25 °C. The supernatant (3 mL) was injected into a cuvette to measure particle size. Specifically, the laser light hits the particle and generates scattered light, and the detector measures the intensity change of the scattered light to calculate the particle size. The signal range is 1 nm–10 µm. All measurements were repeated three times (*n* = 3).

### 2.4. Microstructure Analysis

The microstructure of plant PIH was determined by transmission electron microscopy (TEM) using the method of Wang et al. [29]. A formvar-carbon coated 300-mesh copper grid was used as the TEM grid. For specimen preparation, 10 μL of the sample suspension was dropped on the TEM grid and the excess sample was blotted after 3 min. Then, uranyl acetate was added to the sample solution for positive staining and the excess was blotted after 1.5 min. Next, the TEM specimens were dried in a vacuum dryer and TEM images of the samples were taken using a JEM-1400 microscope (JEOL Co. Ltd., Tokyo, Japan) operating at 100 keV. The statistics of the observed gel size distribution were analyzed analyzed using Lispix, a public domain image analysis program (written by David S. Bright, Microanalysis Research Group, NIST; web page: https://www.nist.gov/services-resources/software/lispix, accessed on 14 April 2021). All measurements were repeated three times (*n* = 3).

### 2.5. Cell Culture

The mouse skeletal muscle cell line (C2C12) (BCRC 60083) was purchased from the Food Industry Research and Development Institute (Hsinchu, Taiwan). C2C12 were cultivated in high-glucose Dulbecco’s modified Eagle’s medium (DMEM, Gibco, Thermo Fisher Scientific, Inc., Waltham, MA, USA) containing 10% fetal bovine serum. The cells were incubated in an incubator at 95% relative humidity, 5% CO_2_ and 37 °C. After the cells grew to 70–80% confluence, the culture medium was changed to 2% horse serum DMEM and replaced every 2 days thereafter. After 6 days of differentiation, multinucleated myotubes were formed.

### 2.6. Cell-Based Antioxidant Assay

To evaluate the protective effects of plant PIH on H_2_O_2_-induced oxidative injury in skeletal muscle cells, cell viability was determined using a colorimetric 3-(4,5-dimethylthiazol-2-yl)-2,5-diphenyltetrazolium bromide (MTT) assay according to the method of Bahuguna et al. [30]. Differentiated C2C12 cells were seeded in 96-well plates at a density of 0.5–1.0 × 10^4^ cells/well and pretreated with various concentrations of plant PIH (25–500 μg/mL) and testosterone (1 µM) in serum-free medium for 24 h. Aspirated the treatments and further treated with 0.2 mM H_2_O_2_ for 1 h. Diluted MTT solution (1 mg/mL) was added to each well (100 μL/well) and for 2 h, the crystallizations were dissolved in DMSO (100 μL/well). The absorbance was measured on an Epoch Microplate Spectrophotometer (BioTek, VT, USA) at 570 nm, with a reference wavelength at 630 nm. C2C12 cells were also seeded in 6-well plates at a density of 1 × 10^5^ cells/well, fixed in methanol and then stained with crystal violet dye. The color intensity quantification was evaluated by ImageJ software (version 1.52, National Institute of Health, Bethesda, MD, USA). Each sample was analyzed in triplicate.

### 2.7. Statistical Analysis

Values are presented as the mean ± standard deviation (SD), and the difference among group means was analyzed using GraphPad Prism 6.0 (GraphPad Software, San Diego, CA, USA). The Student’s *t*-test was used to analyze the difference between two groups, and one-way analysis of variance (ANOVA) was used for data of more than two groups. Tukey’s post hoc test was used for post-test analysis. A *p*-value lower than 0.05 indicated statistical significance between two groups.

## 3. Results and Discussion

### 3.1. MWs Distributions

**Figure 1:**
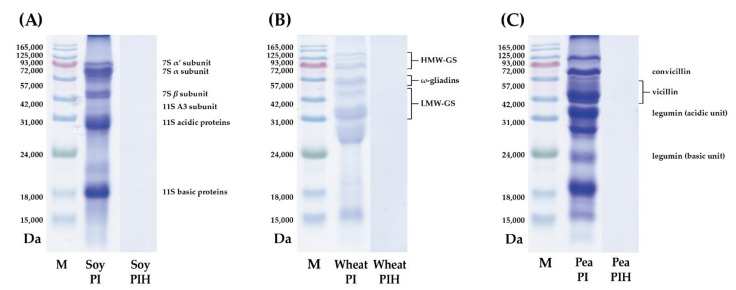
SDS-PAGE analysis of plant PI and PIH samples. (**A**) Soy PI and soy PIH (**B**) Wheat PI and wheat PIH (**C**) Pea PI and pea PIH. PI: protein isolate. PIH: protein isolate hydrolysate. M: MW standard.

**Figure 2:**
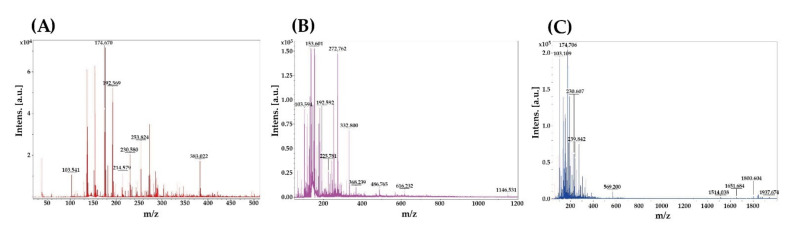
MALDI-TOF-MS analysis of plant PIH samples. (**A**) Soy PIH (**B**) Wheat PIH (**C**) Pea PIH. PIH: protein isolate hydrolysate.

To investigate the protein profile of plant PI after enzymatic hydrolysis, the MWs of three plant PI and three plant PIH were analyzed separately by SDS-PAGE. The storage proteins in soy PI are mainly glycinin globulin (11S) and β-conglycinin globulin (7S) [31]. As shown in Figure 1A, soy PI contains two proteins, 7S (45–75 kDa) and 11S (18–38 kDa). This result is in accordance with a study published by Hsiao et al. [32]. As shown in Figure 1B, wheat PI contains three proteins, HMW-GS (70–100 kDa), ω-gliadins (55 kDa) and LMWGS (30–45 kDa) as previously described by DuPont et al. [33]. The storage proteins of pea PI are legumin (11S), vicilin (7S) and convicilin [34]. As shown in Figure 1C, four types of proteins were determined for pea PI, namely convicilin (65 kDa), vicillin (45 kDa), legumin acidic unit (38 kDa) and legumin basic unit (19–22 kDa). This result is consistent with that in the study published by Opazo-Navarrete et al. [35]. However, no protein bands were observed in the SDS-PAGE analysis of the three plant PIH samples, indicating plant PI can be hydrolyzed to plant PIH by peptide bond cleavage. Li et al. [36] proposed that the MWs of protein hydrolysis products were lower than those of natural proteins. Moreover, according to Karamać and Rybarczyk [37], the area of protein bands decreases or even partially disappears with a higher degree of protein hydrolysis. Therefore, the MWs of three plant PIH samples were further analyzed by MALDI-TOF-MS: soy PIH MWs < 400 Da, wheat PIH MWs < 1200 Da and pea PIH MWs < 2000 Da (Figure 2). Our results show that proteins can be broken down into smaller peptides by enzymatic hydrolysis.

### 3.2. Particle Size Analysis

**Figure 3:**
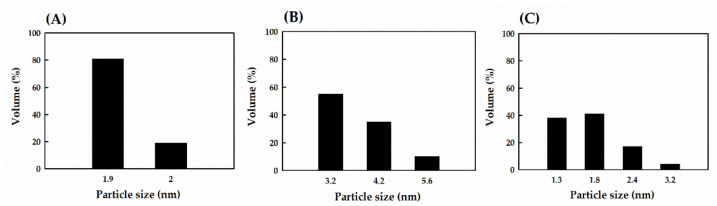
Particle size analysis of plant PIH samples. (**A**) Soy PIH (**B**) Wheat PIH (**C**) Pea PIH. PIH: protein isolate hydrolysate.

Many studies have shown that enzymatic hydrolysis is an effective tool for preparing soy protein nanoparticles [38,39]. To investigate the size of plant PIH particles, a 90Plus Nanoparticle Size Analyzer was used in this study. The average yields of soy PIH at 1.9 and 2.0 nm were 81% and 19%, respectively (Figure 3A); the average yields of wheat PIH at 3.2, 4.2 and 5.6 nm were 55%, 35% and 10% (Figure 3B); and the average yields of pea PIH at 1.3, 1.8, 2.4 and 3.2 nm were 38%, 41%, 17% and 4%, respectively (Figure 3C). The results are similar to those of Chang et al. [40]. Nicklas et al. [41] suggested that collagen nanoparticles of different sizes could be obtained with different hydrolysis times (18–168 h). In summary, enzymatic hydrolysis can modify the structure of proteins and reduce the particle size.

### 3.3. Microstructure Analysis

Midelfort and Wittrup [42] reported proteins form highly specific and structured complexes via several self-assembly methods. These protein particles are formed by aggregation and their size may span multiple orders of magnitude, from oligomers (tens of nanometers) to visible aggregates (hundreds of micrometers). Shen et al. [39] proposed that the protease used for hydrolysis, the substrate protein, the degree of hydrolysis and even the method of enzyme deactivation had a profound effect on the assembly of hydrolysis products.

**Figure 4:**
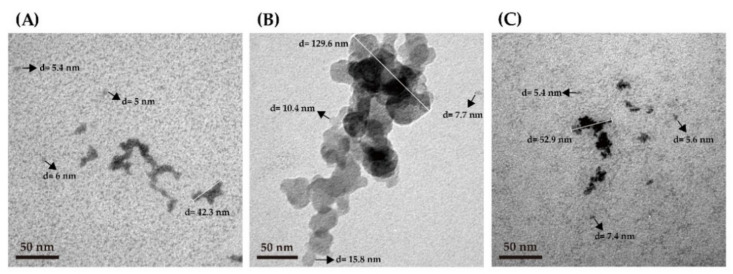
TEM analysis of plant PIH samples. (**A**) Soy PIH (**B**) Wheat PIH (**C**) Pea PIH. PIH: protein isolate hydrolysate.

Sung et al. [43] indicated that TEM was particularly useful for detecting and characterizing the microstructure of aggregates in the submicron range. Therefore, we analyzed the microstructure of three plant PIH particles by TEM. As shown in Figure 4, the particle size of soy PIH was about 5–6 nm with a microstructure presented as aggregated nanoparticles with a length of about 43 nm (Figure 4A), wheat PIH particles were about 7.7–15.8 nm with an aggregated nanoparticle microstructure of about 130 nm long (Figure 4B) and the size of pea PIH particles was about 5.4–7.4 nm with a microstructure of aggregated nanoparticles of about 53 nm in length (Figure 4C). Klompong et al. [44] reported the use of enzymatic hydrolysis in the modification of protein structures, which resulted in small peptides as nanoparticles with different sizes. Therefore, the nanoparticle size and TEM analyses of PPIH were conducted. Our results indicated that the nanoparticle sizes of soy PIH, wheat PIH and pea PIH were 1.9–2.0 nm, 3.2–5.6 nm and 1.3–3.2 nm, respectively (Figure 3). Our previous results showed that the average yields of potato PIH at 1.3, 1.8, 2.4 and 3.2 nm were 50%, 35%, 12% and 3%, respectively [40]. We also noticed that the TEM analysis showed that the particle size of soy PIH, wheat PIH and pea PIH particles were 5.0–42.3, 7.7–129.6 and 5.4–52.9 nm with a microstructure that presented as aggregated nanoparticles. This observation indicates that the particle sizes of soy PIH, wheat PIH and pea PIH obtained from TEM analysis were higher than the nanoparticle size. However, the particle aggregation of plant PIH may occur during the drying process before TEM analysis. For TEM analysis, the plant PIH suspension was dropped on the TEM grid, and the TEM specimens were dried in a vacuum dryer. This process may cause the aggregated plant PIH to form larger aggregates and show an aggregated nanoparticle structure.

### 3.4. Cell-Based Antioxidant Activities

**Figure 5:**
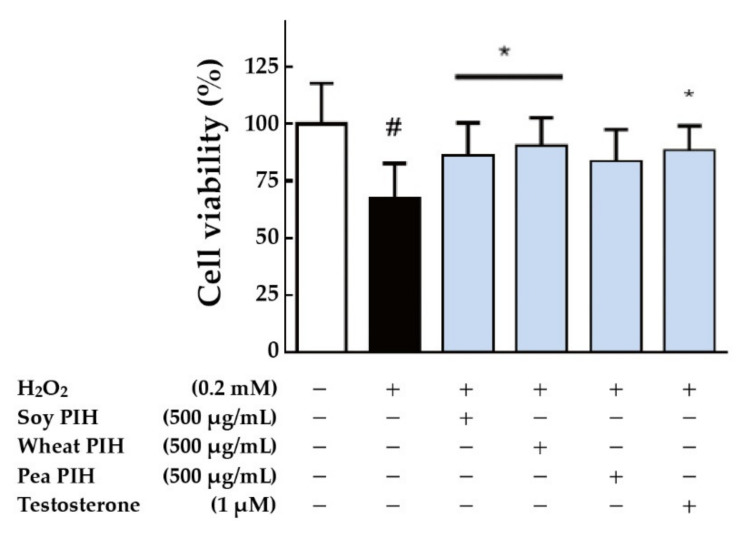
Effect of plant PIH samples on the cell viability of C2C12 cells. C2C12 myoblasts (1 × 10^4^ cells) were cultured in 96 well plate used 2% horse serum DMEM to differentiate to myotube. After 6 days of differentiation, pretreated with 500 μg/mL plant PIH for 24 h and then stimulated with 0.2 mM H_2_O_2_ for 1 h. # *p* < 0.05 versus control (white column), * *p* < 0.05 versus H_2_O_2_ only treated cells (Black column). PIH: protein isolate hydrolysate.

Protein hydrolysates have many health benefits including antioxidant effects [9]. Recent studies showed that plant protein hydrolysates including potato PIH and soy PIH act as free radical scavengers or antioxidants [45]. The treatment of plant PIH showed no toxicity to the C2C12 myotube (Supplementary Figure S1). To investigate the antioxidant effects of plant PIH in vitro, a mouse skeletal muscle cell line (C2C12) was treated with three types of plant PIH (soy, wheat and pea) for 24 h and then subjected to oxidative damage by H_2_O_2_ (0.2 mM). The cell viability was analyzed by MTT test. As shown in Figure 5, the direct H_2_O_2_ treatment (black column) significantly reduced the cell viability of C2C12 compared with the control group (white column) (*p* < 0.05). Pre-treatment with soy PIH or wheat PIH (500 μg/mL) had a significant protective effect on cells compared with the group treated with H_2_O_2_ directly (black column) (*p* < 0.05). The administration of testosterone (1 μM) also significantly prevented oxidative damage caused by H_2_O_2_ (*p* < 0.05). These results showed that soy PIH and wheat PIH were effective at preventing oxidative damage to C2C12 caused by H_2_O_2_.

**Figure 6:**
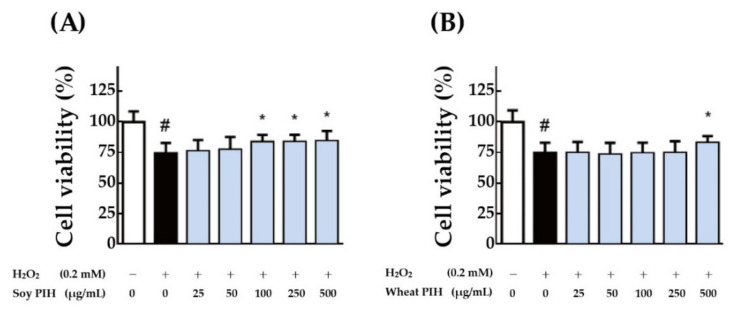
Effects of various concentrations with plant PIH samples on the viability of C2C12 cells. (**A**) Soy PIH (**B**) Wheat PIH. C2C12 myoblasts (1 × 104 cells) were cultured in 96-well plate used 2% horse serum DMEM to differentiate to myotube. After 6 days of differentiation, pretreated with 25–500 μg/mL soy or wheat PIH for 24 h and then stimulated with 0.2 mM H2O2 for 1 h. # *p* < 0.05 versus control, * *p* < 0.05 versus H2O2 only treated cells (Black column). PIH: protein isolate hydrolysate.

**Figure 7:**
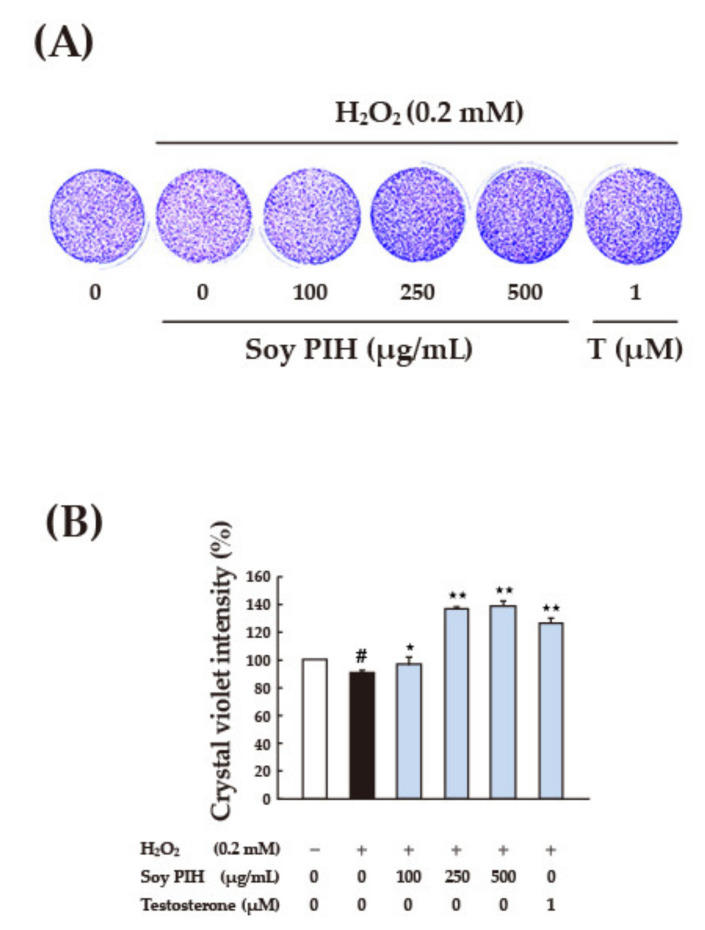
Effect of various concentrations of soy PIH on the cell morphology of C2C12 cells. (**A**) Cell morphology (**B**) Crystal violet intensity. C2C12 myoblasts (1 × 10^5^ cells) were cultured in 96 well plate used 2% horse serum DMEM to differentiate to myotube. After 6 days of differentiation, pretreated with 100–500 μg/mL soy PIH for 24 h and then stimulated with 0.2 mM H_2_O_2_ for 1 h. # *p* < 0.05 versus control, * *p* < 0.05 or ** *p* < 0.01 versus H_2_O_2_ only treated cells (Black column). PIH: protein isolate hydrolysate.

**Figure 8:**
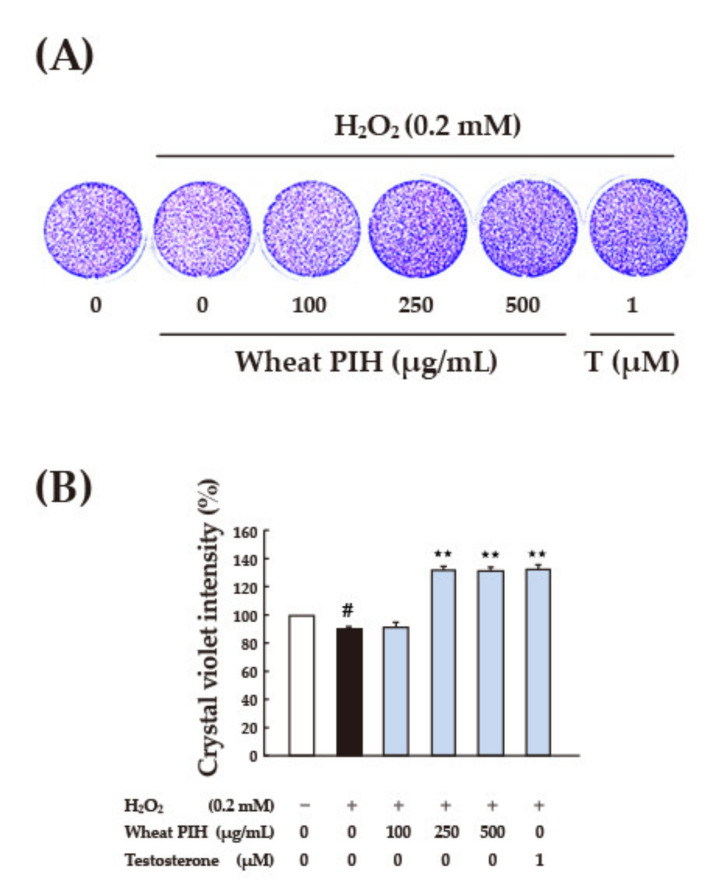
Effect of various concentrations of wheat PIH on the cell morphology of C2C12 cells. (**A**) Cell morphology (**B**) Crystal violet intensity. C2C12 myoblasts (1 × 10^5^ cells) were cultured in 96 well plate used 2% horse serum DMEM to differentiate to myotube. After 6 days of differentiation, pretreated with 100–500 μg/mL wheat PIH for 24 h and then stimulated with 0.2 mM H_2_O_2_ for 1 h. # *p* < 0.05 versus control, ** *p* < 0.01 versus H_2_O_2_ only treated cells (Black column). PIH: protein isolate hydrolysate.

A plant PIH dose that had been proven effective was tested. After 24 h of pretreatment with soy PIH and wheat PIH at different concentrations (25–500 μg/mL), H_2_O_2_ (0.2 mM) was added, and the treatment lasted for 1 h. The results indicated that pretreatment with soy PIH at doses of 100–500 μg/mL had a significant protective effect on cells (*p* < 0.05) compared with the group treated with H_2_O_2_ directly (black column), (Figure 6A). Wheat PIH at a dose of 500 μg/mL was required to show a significant protective effect (*p* < 0.05) (Figure 6B). Crystal violet staining was performed on cells treated with various doses (100–500 μg/mL) of soy PIH and wheat PIH; however, no obvious difference or change was observed from the stained images (Figure 7A and Figure 8A). Image quantification showed H_2_O_2_ decreased the number of C2C12 cells and that pretreatment with soy PIH (100–500 μg/mL) (Figure 7B) and wheat PIH (250–500 μg/mL) (Figure 8B) had a protective effect. The administration of testosterone (1 µM) also significantly prevented oxidative damage caused by H_2_O_2_. The above results showed that plant PIH had a protective effect against H_2_O_2_-induced oxidative damage to C2C12, and the magnitude of the antioxidant effect was in the order of soy PIH > wheat PIH.

Skeletal muscle has a unique capability to enhance the rate of oxygen consumption during contraction [46]. During intense exercise, high oxygen consumption rates in skeletal muscle can lead to incomplete oxygen reduction and electron leakage from the electron transport chain, resulting in the production of ROS, and excess ROS can cause further cell and tissue damage [47,48]. Pronsato et al. [49] reported that testosterone could protect C2C12 muscle cells against apoptosis induced by H_2_O_2_ through the apoptotic intrinsic pathway. Furthermore, La Colla et al. [50] suggested that testosterone could protect mitochondria against oxidative stress in skeletal muscle cells. According to our previous study [40], potato protein isolate hydrolysate demonstrated ABTS radical scavenging activity capable of protecting C2C12 cells from H_2_O_2_ oxidation. Therefore, these three plant PIHs may also show ABTS radical scavenging activity and protect C2C12 muscle cells against apoptosis induced by H_2_O_2_. Through enzymatic hydrolysis, proteins release many bioactive peptides that have antioxidant capacity and may play an important role in human health [9]. According to Hwang et al. [22], black bean peptide (40 μg/mL) protected skeletal muscle cells (C2C12) from oxidative damage by H_2_O_2_. Kerasioti et al. [51] reported that whey protein (0.5 mg/mL) reduced ROS content in skeletal muscle cells (C2C12) and had a protective effect against the oxidative stress caused by ROS. The results of this study indicate that soy PIH and wheat PIH have protective effects against H_2_O_2_-induced oxidative damage to C2C12. This suggests plant PIH have antioxidant activities and can protect cells from free radical damage. In this study, testosterone promoted skeletal muscle synthesis and was used as a positive control. Testosterone is the main hormone that interacts with internal androgen receptors to induce the growth, recovery and remodeling of muscle tissues [52] Our study confirmed the antioxidant activity of soy PIH and wheat PIH, comparable to testosterone effect. Soy PIH had a high antioxidant effect and might be used as a peptide with antioxidant activity (*MW* < 400 Da). Shen et al. [39] also reported that soy protein isolate hydrolysate (SPIH) generated by enzymatic hydrolysis simultaneously conferred improved antioxidant activity, and the enhanced surface hydrophobicity together with the specific hydrolysis of soy protein isolate into polypeptides endowed SPIH with a superior anti-oxidative capacity. Therefore, soy PIH might be useful as an antioxidant in medical applications and as a quality source of amino acids in vegetarian and senior-friendly foods.

## 4. Conclusions

This study confirmed that the MWs and particle size of plant PI can be reduced by enzymatic hydrolysis. All three types of plant PIH tested in this study are in the nanoscale with the characteristics of aggregating to form larger aggregates. In terms of the viability of C2C12 cells subjected to H_2_O_2_-induced oxidative damage, the protective effect of soy PIH was superior to that of wheat PIH and pea PIH, indicating soy PIH has high antioxidant activity, which might be a suitable antioxidant peptide for medical applications.

## Data Availability

The data presented in this study are available from the corresponding author.

## Supplementary Materials

The following are available online at https://www.mdpi.com/article/10.3390/nano11061509/s1: Figure S1. Toxicity effect of PIH on C2C12.
