# Supplementary material for: Antioxidative Activity of Soy, Wheat and Pea Protein Isolates Characterized by Multi-Enzyme Hydrolysis
Source: Nanomaterials (Basel). 2021 Jun 7;11(6):1509. doi: 10.3390/nano11061509 (PMC8227270; doi:10.3390/nano11061509)
Supplement: Supplementary file 1 [file nanomaterials-11-01509-s001.zip › nanomaterials-1203480-supplementary.pdf]

nanomaterials

Article

Antioxidative Activity of Soy, Wheat and Pea Protein Isolates Characterized by Multi-Enzyme Hydrolysis

Chiung-Yueh Chang [1], Jinn-Der Jin [2], Hsiao-Li Chang [2], Ko-Chieh Huang [1], Yi-Fen Chiang [1], Mohamed Ali [3] and Shih-Min Hsia [1,4,5,6,*]

1 School of Nutrition and Health Sciences, College of Nutrition, Taipei Medical University, Taipei 110, Taiwan; d507104001@tmu.edu.tw (C.-Y.C); a910241@gmail.com (K.-C.H); yvonne840828@gmail.com (Y.-F.C); bryanhsia@tmu.edu.tw (S.-M.H)
2 GeneFerm Biotechnology Co., Ltd., Tainan 741, Taiwan; jin168@geneferm.com (J.-D.J); gracechang@geneferm.com (H.-L.C)
3 Clinical Pharmacy Department, Faculty of Pharmacy, Ain Shams University, Cairo 11566, Egypt; mohamed.aboouf@pharma.asu.edu.eg
4 Graduate Institute of Metabolism and Obesity Sciences, College of Nutrition, Taipei Medical University, Taipei 110, Taiwan
5 School of Food and Safety, Taipei Medical University, Taipei 110, Taiwan
6 Nutrition Research Center, Taipei Medical University Hospital, Taipei 110, Taiwan
* Correspondence: bryanhsia@tmu.edu.tw; Tel.: +886-2-2736-1661

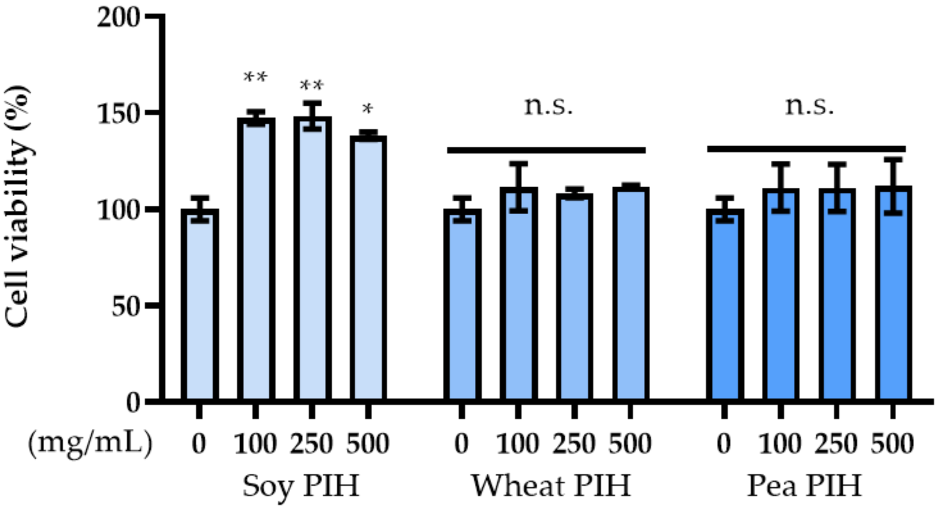

Figure S1. Toxicity effect of PIH on C2C12. C2C12 myoblasts (1×10^4 cells) were cultured in 96-well plates using 2% horse serum DMEM to differentiate the myotubes. After 6 days of differentiation, the samples were treated with different dosages of plant PIH for 24 hours * $p < 0.05$; **, $p < 0.01$ versus 0 mg/mL cells. PIH: protein isolate hydrolysate.
